# Naïve B cells followed by aquaporin-4 antibodies characterise the onset of neuromyelitis optica: evidence from stem cell transplantation

**DOI:** 10.1136/jnnp-2022-328982

**Published:** 2022-05-23

**Authors:** Peter McNaughton, Rebecca Payne, Sophia Michael, Timothy Leahy, Alexander Nicols, Andrew Fower, Sophie Hambleton, Ki Pang, Andrew Gennery, Sarosh R Irani

**Affiliations:** 1 Paediatric Immunology, Newcastle upon Tyne Hospital Trusts, Newcastle upon Tyne, UK; 2 Department of Paediatrics, Queensland Children's Hospital, South Brisbane, Queensland, Australia; 3 Nuffield Department of Clinical Neurosciences, University of Oxford, Oxford, UK; 4 Department of Neurology, Oxford University Hospitals NHS Foundation Trust, Oxford, UK; 5 Children's Health Ireland at Crumlin, Trinity College Dublin, Dublin, Ireland; 6 Nuffield Department of Clinical Neurosciences, John Radcliffe Hospital, Oxford, UK; 7 Department of Paediatric Neurology, Newcastle upon Tyne Hospitals NHS Foundation Trust, Newcastle upon Tyne, UK

**Keywords:** IMMUNOLOGY, NEUROIMMUNOLOGY, PAEDIATRIC NEUROLOGY

## Introduction

Neuromyelitis optica spectrum disorders (NMOSDs) are mediated by antibodies directed against the extracellular domain of aquaporin-4 (AQP4). These antibodies form a key pillar in diagnostic criteria for NMOSD.^1^ Yet, the immunological mechanisms underlying the generation of AQP4 antibodies during disease initiation are incompletely understood, principally because this is an asymptomatic period. To date, AQP4 antibodies and symptomatic NMOSD are known to develop several years after myasthenia gravis, typically post-thymectomy,^2^ or in the context of bone marrow transplantation.^3^ These examples suggest the immunopathogenesis of symptomatic NMOSD typically requires many years to mature.

The duration and nature of the immune response maturation can provide insights into the cellular processes responsible for AQP4 antibody production, in particular the potential relevance of long-lived plasma cells versus germinal centre reactions.^4 5^ Hence, the fundamental immunopathogenesis may inform the rational selection of targeted immunotherapeutics.^4 5^

Here, we describe a patient who developed post-transplant NMOSD, and capture the key period of acute clinico-serological disease conversion with serial biological samples. The findings revealed herein provide several unique insights into the immunopathogenesis of NMOSD.

## Materials and methods

*Phenotype and patient samples.* Clinical and radiology data collection was prospectively gathered, along with serial blood samples (both cells and serum), and archived for research purposes.

*AQP4-antibodies*. Live cell-based assays were performed, with minor modifications from published protocols.^6^ In brief, HEK293T cells were transfected with cDNA encoding full-length AQP4 and, while live, labelled with patient IgG or IgM which, after fixation, were detected with isotype-specific secondary antibodies (product numbers 709-585-098, Jackson labs, and A-21216, Thermofisher, respectively). Prior to AQP4-IgM detection, IgGs were fully depleted with protein G beads. All positive results were titrated to endpoint dilutions.

*B cell populations*. From liquid nitrogen archived whole blood, mass cytometry immunophenotyped several populations including B cells (details in online supplemental data). Naïve B cells were defined as CD19^+^CD20^+^CD27^−^IgD^+^.

10.1136/jnnp-2022-328982.supp1 Supplementary data

## Results

### Clinical features

A boy (between 1 and 2 years of age) with *STAT3* gain-of-function mutation received a matched unrelated donor peripheral blood stem cell transplant to treat severe refractory multisystem autoimmune disease, including neonatal giant cell hepatitis and complete lipodystrophy.

**Figure 1:**
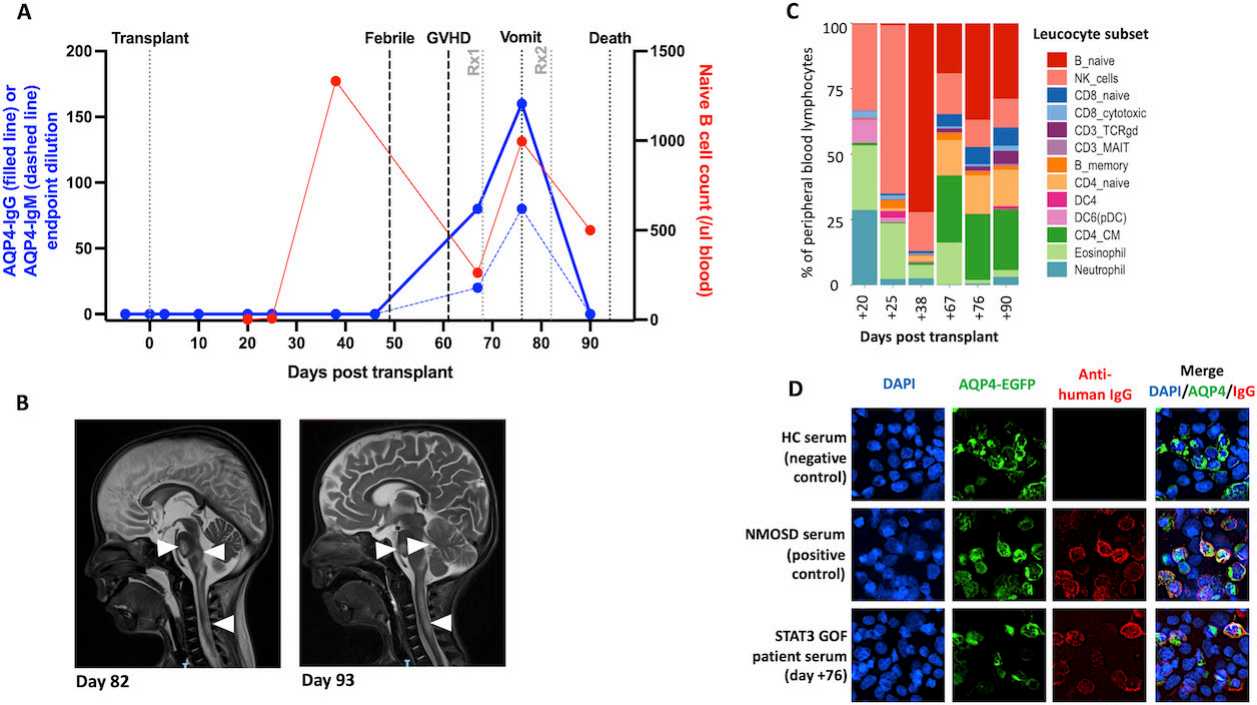
Time course of cellular immune reconstitution: naïve B cell repopulation, AQP4 antibody generation and imaging changes following stem cell transplant. (A) Time course of peripheral blood naïve B cell counts (red, right y-axis), and serum AQP4-IgG (blue solid line, left y-axis) and AQP4-IgM (blue dashed line, left y-axis) endpoint dilutions. The first sample obtained was preconditioning. Treatment 1 (Rx1, grey) was methylprednisolone (30 mg/kg); treatment 2 (Rx2, grey) was methylprednisolone (30 mg/kg), plasmapheresis and alemtuzumab (0.2 mg/kg). Vomit=onset of vomiting. (B) Sagittal T2-weighted MRI shows regions of inflammation in the brainstem and cervical cord (arrowheads) at day 82 and day 93 post-transplant. (C) Proportions of peripheral blood leucocyte subsets as measured by mass cytometry (Cytof-Helios). Annotated subsets include naïve B cells (CD19^+^CD20^+^CD27^−^IgD^+^); memory B cells (CD19^+^CD20^+^CD27^+^), natural killer (NK), CD3^+^ gamma delta T cells (gdTCR), CD3^+^ mucosal-associated invariant T cells (MAIT), dendritic cells (DC, including plasmacytoid DCs; pDC) and both naïve/central memory CD4^+^ T cells (CD4_CM). (D) AQP4-antibody live cell-based assay. AQP4-IgGs (red) from a serum of a patient with neuromyelitis optica spectrum disorder (NMOSD) binds to the surface of live AQP4-expressing HEK293T cells (middle panel), with similar reactivity demonstrated by the STAT3 gain-of-function (GOF) patient serum (bottom panel). Healthy control (HC) serum (top panel) shows no detectable binding to these cells. AQP4 tagged to EGFP (AQP4-EGFP, green); DAPI nuclear staining (blue). Images taken at ×40 magnification. AQP4, aquaporin-4; DAPI, 4′,6-diamidino-2-phenylindole; EGFP, enhanced green flourescent protein; GVHD, graft-versus-host disease.

After an unremarkable early post-transplant course, on day 49 he developed a fever and respiratory distress, with no infective cause identified (figure 1A). On day 61, oedema, rash and diarrhoea led to a diagnosis of graft-versus-host disease (GVHD), confirmed on upper gastrointestinal tract biopsy and treated with methylprednisolone (2 mg/kg) from day 68. Subsequently, on day 76, he developed severe vomiting, initially considered secondary to progressive GVHD. However, after 1 week he had slow pupillary reactions, left-sided weakness, a decreased level of consciousness and apnoea. MRI showed T2 hyperintense lesions predominantly affecting the pons, medulla, area postrema and cervical cord (figure 1B), with optic nerve sparing. Serum AQP4-IgG was detected with normal total immunoglobulin levels. He was diagnosed with NMOSD and treated aggressively with 30 mg/kg methylprednisolone, plasmapheresis and alemtuzumab (0.2 mg/kg×5 doses). On day 93, he developed labile blood pressure and fixed-dilated pupils. Repeat MRI showed brainstem lesion extension plus new bithalamic involvement (figure 1B). The neurological disease was considered irreversible and respiratory support withdrawn on day 94.

### Laboratory findings

Retrospective live cell-based assays showed the *de novo* appearance of serum AQP4 antibodies (1:80 endpoint dilution) on day 67, with levels which rose to 1:160 by day 76 (figure 1). After confirmed depletion of IgG, these two samples additionally showed AQP4-IgM reactivities (1:40 and 1:80 endpoint dilutions, respectively). No other samples showed AQP4-IgM or AQP4-IgG. Mass cytometry analysis revealed that these serological findings were preceded by a striking expansion of the naïve B lymphocyte population, between days 25 and 38, rising from 0.8% to 72% of all leucocytes (figure 1A–C). This time course represents a highly accelerated reconstitution of the naïve B cell compartment, which is usually delayed until >6 months post-transplant.^7^ Genotyping on day 83 (a comparison of donor and recipient DNA using PowerPlex 16 HS system) revealed that 70% of CD19^+^ cells were donor derived (30% were from the recipient); whereas none of the residual CD3^+^ T cells and only 21% of myeloid cells were donor derived.

## Discussion

This tragic case provides a unique opportunity to observe a *de novo* human autoimmunisation directed against AQP4. Below, we synthesise longitudinal clinical, cellular and serological observations from this distinctive case to hypothesise mechanisms of AQP4 antibody synthesis, with both clinical and therapeutic relevance.

The temporal dynamics of this human autoimmunisation identified the generation of AQP4 antibodies over just a few weeks, early after stem cell transplantation and far more acutely than documented in two different clinical scenarios.^2 3^ An unusually sharp ~100-fold rise in naïve B cells occurred prior to generation of AQP4 antibodies. This time course may reflect the exit of donor antigen-inexperienced B cells from the bone marrow (70% of the B cells were donor derived) and their subsequent maturation towards precursors of the serum AQP4 antibodies.

Around 1 month later, both *de novo* serum AQP4-IgG and IgMs were observed and temporally coincided with the development of symptomatic NMOSD. The concurrent AQP4-IgG and IgMs suggest an acute immunisation in this patient (akin to that observed in many infections), and support a germinal centre-based generation of AQP4 antibodies. This germinal centre activity may be fuelled by the reconstituting naïve B cells which, in patients with NMOSD, have been observed to both carry AQP4 reactivities and show deranged regulatory properties.^6 8^ Hence, prevention of naïve B cell reconstitution, for example, with anti-CD19 and/or anti-CD20 drugs, may offer an important therapeutic target which represents a potential precursor to relapses in NMOSD.^9^ In further support of this mechanism, a few weeks is likely too short a duration to generate a significant population of human long-lived plasma cells. Yet, it remains possible that the AQP4-IgG generation resulted from incomplete depletion of plasma cells prior to transplantation.

STAT3 is a pleotropic transcription factor expressed by the NMOSD-associated Th17 T cell subset,^10^ which also drives the differentiation of T follicular cells and inhibits the generation of T regulatory cells. Therefore, it may be that disordered STAT3 signalling, particularly from the recipient’s residual T cells, could be implicated in the pathogenesis of their NMOSD.^3^

In summary, by detailing a case with an early, severe neurological complication after stem cell transplantation, we provide an opportunity to observe *in vivo* the development of AQP4 antibodies. Our data support a role for naïve B cells and germinal centres in the initiating pathogenesis of NMOSD. This conclusion has important implications for understanding disease pathogenesis and selecting optimal therapeutics.
