# Supplementary material for: Naïve B cells followed by aquaporin-4 antibodies characterise the onset of neuromyelitis optica: evidence from stem cell transplantation
Source: J Neurol Neurosurg Psychiatry. 2022 May 23;93(11):1234–6. doi: 10.1136/jnnp-2022-328982 (PMC9606486; doi:10.1136/jnnp-2022-328982)
Supplement: Supplementary data [file jnnp-2022-328982supp001.pdf]

Supplementary data

Mass cytometry.
Briefly, white blood cells (WBCs) were prepared from lysed whole blood and live/dead stained using Cell-ID cisplatin (5µM, Fluidigm, 201064). After washing, WBCs were surface stained, at room temperature, with primary (30 minutes) and secondary (60 minutes) antibodies, washed and fixed using 1.6% formaldehyde for 1 hour. Fixed cells were washed and cryopreserved in freezing media (FBS + 10% DMSO). Thereafter, thawed samples were washed and permeablized using 0.1% Triton X-100, washed and stained intracellularly overnight at 4°C. Finally, cells were retrieved, washed and intercalated for 1 hour (Ir-125µM, 1:1000, Fluidigm, 201192A) in Maxpar fix/perm buffer (Fluidigm, 201067) and acquired immediately on a Helios mass cytometer platform (Fluidigm). Normalized and bead excluded FCS files were analysing within a R pipeline, populations were clustered and visualised using *FlowSOM* and *ConsensusCentrePlus* wrapped within *CATALYST* package.

B populations were determined using antibodies against CD19, CD20, CD27 and IgD.
